# Multicentric randomized clinical trial to evaluate the long-term effectiveness of a motivational intervention against smoking, based on the information obtained from spirometry in primary care: the RESET study protocol

**DOI:** 10.1186/s12875-016-0415-1

**Published:** 2016-02-04

**Authors:** Francisco Martin-Lujan, Antoni Santigosa-Ayala, Josep-Lluis Piñol-Moreso, Mar Sorli-Aguilar, Gemma Flores-Mateo, Jordi Bladé-Creixenti, Josep Basora-Gallisà, Rosa Sola-Alberich, for the RESET Study Group investigators

**Affiliations:** Study Group on Respiratory Tract Diseases (GEPAR), Institut Universitari d’Investigació en Atenció Primària Jordi Gol (IDIAP Jordi Gol), Barcelona, Spain; School of Medicine and Health Sciences, Universitat Rovira i Virgili, Tarragona, Spain; CAP Sant Pere - Institut Català de la Salut, C/Cami de Riudoms, 53-55, Tarragona, Reus-43203 Spain; Primary Healthcare Research Support Unit Tarragona-Reus, Institut Universitari d’Investigació en Atenció Primària Jordi Gol (IDIAP Jordi Gol), Reus, Spain

**Keywords:** Spirometry, Tobacco smoking, Motivational intervention, Primary health care

## Abstract

**Background:**

Spirometry is the recommended method of evaluating pulmonary function when respiratory disease is suspected in smokers. Nonetheless, no evidence exists of the usefulness of information obtained from this test as a motivational strategy for smoking cessation. The primary objective of this study is to evaluate the effectiveness of a motivational intervention based on spirometry results in achieving long-term smoking cessation.

**Methods/Design:**

We propose a multicenter randomized clinical trial in the primary care setting. **Study subjects**: We will recruit active smokers of both sexes, aged 35-70 years, with a cumulated smoking habit exceeding 10 packs/year and who consult for any reason with their primary care physician in the 20 health centers in the province of Tarragona (Spain). Patients with a history of lung disease or who have undergone exploratory measures of pulmonary function in the preceding 12 months will be excluded. All patients who agree to participate will provide signed informed consent prior to their inclusion. A total of 1000 smokers will be consecutively randomized to a control or intervention group (1:1). **Intervention:** Participants in both groups will receive brief (5-minute) health counseling, in accordance with usual clinical practice. In a consultation lasting about 15 minutes, participants in the intervention group will also receive detailed, personalized information about the results of a spirometry test and about their lung age compared with their chronological age. Both groups will be followed up for 12 months. **Main variables and analysis:** The main variable will be sustained smoking abstinence at 12 months after the intervention, as confirmed by CO breath testing and urine cotinine test. Results will be analyzed based on intention to treat, using the chi-square test and logistical regression if necessary to adjust for confounding variables.

**Discussion:**

We expect the rate of prolonged smoking abstinence in the intervention group will be at least 5 % higher than in the control group. If this strategy proves effective, it could easily be included in the health promotion activities offered in primary care settings.

**Trial registration:**

ClinicalTrials.gov Identifier: NCT02153047. Registered on 28/05/2014

## Background

Diseases related to smoking frequently motivate patient visits to health care settings in general and primary care in particular. The magnitude of this health problem has been widely studied and has been cited as the primary preventable cause of mortality and morbidity in industrialized countries [1].

The available data indicate that the life expectancy of habitual smokers is reduced by 15-20 years and approximately half will die as a consequence of their habit [2]. Taking this into account, it is surprising that the detrimental health effects of smoking have so little social impact and that smoking continues to be highly prevalent in our context [3]. The health benefits of smoking cessation are well established. With few exceptions, future risk is reduced when smokers stop smoking and continue to decrease while smoking abstinence is maintained. Progression of smoking-related diseases is also slowed and life expectancy improves by an average of 10 years [2]. For these reasons, anti-smoking efforts in all contexts provide one of the major central points around which many disease prevention strategies revolve [3].

Treatment of the effects of smoking is an important part of clinical practice and all health professionals need to offer help and support to all smokers as part of usual clinical practice [4]. Current guidelines base their recommendations on the “The 5As approach” (ask about tobacco use, advise to quit, assess willingness to make a quit attempt, assist in quit attempt, and arrange follow-up) [5]. According to this model, the health professional who knows the smoker’s motivation can choose the strategy that has shown the greatest effectiveness in each situation. Nonetheless, cigarette smoking is a highly addictive habit that is difficult to give up, and achieving the motivation to quit smoking is always a challenge. The available data suggest that the majority of smokers are interested in quitting [6] and that structured counseling by health care professionals is a motivational element for smoking cessation [7]. However, the rate of successful quit attempts is low and does not usually exceed 10 % [8]. To increase the success rate and accelerate change in smoking habits, it has been suggested that instead of providing patients with general information about the detrimental heath effects of smoking, they should be provided with personalized data on their risk of acquiring a disease related to smoking [9].

Health concerns are among the reasons most often cited as catalysts for smokers to make an attempt to quit smoking [10, 11], and studies suggest that the more a patient is aware of risks and benefits, the greater is their motivation and likelihood of success [12]. Similar to what occurs in smokers who have experienced smoking-related complications, providing information about testing for smoking-related harm or risks to health offers a “teachable moment” that may facilitate behavioral change [13]. In this respect, we can conceptually distinguish three different feedback methods, depending on the approach used to assess risk [14]: the first explores biomarkers of tobacco exposure, such as levels of urine cotinine or exhaled carbon monoxide (eCO); the second gives information about added risk of diseases related to a smoking habit, such as genetic susceptibility to lung cancer; and the third focuses on smoking-related harms such as the presence of arterial plaque detected by echography or decreased pulmonary function quantified by spirometry.

Spirometry is the recommended method of testing pulmonary function to detect restricted respiratory flow in smokers susceptible to chronic obstructive pulmonary disease (COPD) [15]. The use of spirometry has become generalized in primary care because it is a simple, noninvasive procedure that provides valuable information about pulmonary function and risk of premature death [16, 17] that can increase motivation to quit smoking [18]. Some years ago, the Lung Health Study showed that significantly more participants included in an intensive smoking cessation program quit smoking, compared to those included in the usual program [19]. Nonetheless, the experts involved in the most recent report published by the U.S. Preventive Services Task Force did not find sufficient evidence to conclude that the use of spirometry improved rates of smoking abstinence or affected the management of the initial stages of COPD [20].

In recent years, various clinical trials have explored the effect on smoking cessation of providing feedback on spirometry results. The first study, published by Segnan et al [21], included 923 smokers recruited by 44 primary care doctors in Italy. Participants were randomly assigned to 4 different intervention groups: (a) a brief individual intervention, (b) an intensive intervention with 4 follow-up sessions, (c) an intensive intervention and treatment with nicotine gum, and (d) an intensive intervention and spirometry. The proportion of exsmokers at 12-month follow-up was 4.8 %, 5.5 %, 7.5 %, and 6.5 %, respectively, with no significant differences between the groups (RR: 1.19; 95 %CI: 0.62-2.30). Later, Buffels et al [22] published a study that included 215 smokers attending 14 primary care centers in Belgium who were prescribed treatment with nicotine, bupropion, or both and were randomly assigned to receive only a minimal intervention or the intervention along with information about their spirometry results. After 12 months of follow-up, a nonsignificant effect in favor of the spirometry intervention was observed (RR: 1.17; 95 %CI: 0.66-2.06). More recently, Parkes et al [23] published a clinical trial that included 561 smokers recruited in 5 primary care centers in England. All participants were assessed using spirometry and then randomized to receive personalized information about the results obtained, summarized as “lung age” compared to chronological age (intervention group), or to receive a letter reporting the results (control group). After 12 months of follow-up, the rates of punctual smoking abstinence were significantly higher in the intervention group (13.6 % vs 6.4 % in the control group, *p* = 0.005) and these participants had higher probability of successful smoking cessation than those who did not receive personalized spirometry results (RR: 2.12; 95 %CI: 1.24-3.62). Nonetheless, the observed abstinence rate could have been influenced by the intensity of the intervention that was administered (longer period of contact in the intervention group due to verbal reinforcement); it has been shown that abstinence rates are directly proportional to the intensity of the strategy used [24].

In a Cochrane Database of Systematic Reviews article, Bize et al evaluated those three studies and 12 other clinical trials that have used various biomarkers as a motivational strategy for successful smoking cessation [25]. Unfortunately, the designs of the studies included in the review were so heterogeneous that only two pairs of studies were sufficiently similar in methodology and intervention to allow a combined analysis. The authors also found no evidence of significant benefits from the use of eCO measurement or spirometry in primary care to improve success rates for smoking cessation efforts. Therefore, they conclude that, due to the lack of quality evidence, it is not possible to establish definitive recommendations about the effectiveness of most approaches used to evaluate biomedical risk assessment as a means of helping smokers quit smoking. They emphasize a need to improve the methodological quality of future studies. Their review included two active clinical trials with similar methodology, ESPIROTAB and ESPITAP [26, 27], that use spirometry results as a motivational strategy to increase smoking cessation success rates beyond the levels obtained by usual counseling, as in the study by Parkes et al [23]. Nonetheless, this type of study design has been criticized because, strictly speaking, it does not provide independent evidence of the effectiveness of using spirometry, compared to no spirometry; this would require a trial in which spirometry is not used in the control group [28].

The use of spirometry as a motivational tool for smoking cessation continues to be a controversial topic. Some data indicating effectiveness are encouraging, but the scarcity of evidence limits the capacity to establish definitive recommendations about its use.

For all these reasons, further studies are essential, and must use high-quality methodology with a sufficiently large sample and long-term follow-up of at least 12 months to establish a final outcome of prolonged smoking abstinence, confirmed by a biochemical method [25].

## Hypothesis and objectives

### Hypothesis

An intervention based on providing structured, standardized information about the results of spirometry as part of usual clinical practice to encourage patients to quit smoking will achieve improvement of 5 % or more in the rate of prolonged abstinence from smoking at 12 months of follow-up, compared to that obtained by providing only the usual brief counseling to patients who smoke.

### Objectives

The primary objective of this study is to evaluate the effectiveness of providing structured information about the results of spirometry testing to increase the rate of prolonged abstinence from smoking at 12 months after an anti-smoking intervention in primary care patients who smoke but do not have a known respiratory disease.

## Material and methods

### Design

**Fig. 1:**
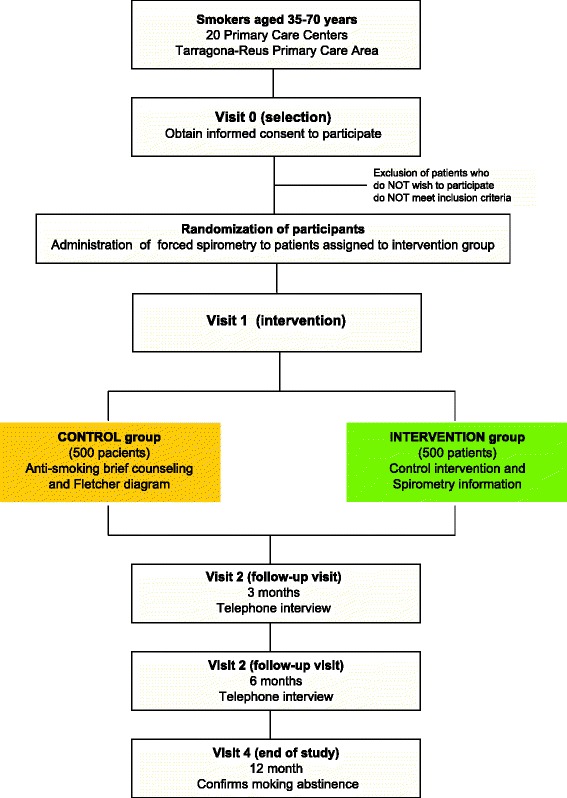
Diagram of the RESET study: process of selection, randomization and follow-up of subjects included in the study

The general framework of the RESET study is shown in Fig. 1 and the activities to be carried out at each visit are detailed in Table 1. This is the second phase of the ESPITAP study (Spanish acronym for “Effectiveness of Smoking Cessation Advice Combined With Spirometric Results in Adult Smokers”), a randomized, controlled, parallel, multicenter clinical trial in smokers with no history of respiratory disease, recruited when they visited a primary care center [27].

**Table 1:** Diagram of activities to be carried out by participants at each visit

	Visit 0	Visit 1	Visit 2	Visit 3	Visit 4
	Selection Inclusion Randomization	Intervention	Follow-up Reminder Intervention	Follow-up Reminder Intervention	End of study
Timeline	Day -30 to -1	Day 0	3 months	6 months	12 months
Procedures					
Informed Consent	X				
Sociodemographic data	X				
Personal history	X				
Basic physical examination	X				
Smoking habit and coximetry	X				
Randomization	X				
Spirometry	X				
Intervention					
Control group: brief health counseling		X			
Intervention group: brief health counseling + information about spirometry results		X			
Measures of effectiveness					
Smoking habit			X	X	X
Confirmation of smoking abstinence (coximetry and urine cotinine)					X

### Setting and study population

The study population will be obtained from patients who consult any primary care center managed by the Catalan Institute of Health in the province of Tarragona. These 20 centers, 12 urban and 8 rural, employ 286 family doctors who care for an adult population (older than 18 years) of 279,637 patients.

### Participant selection

Eligible participants include all patients aged 35 to 70 years who smoke, who consult their primary care team during regular office hours for any reason during the inclusion period, and who meet all inclusion criteria and none of the exclusion criteria, as follows:

- Inclusion criteria: active cigarette smoker with a cumulative habit of more than 10 packs/year. An active smoker is defined as one who acknowledges having smoked regularly during the past 30 days, regardless of the quantity. Cumulative consumption (pack-years) is defined as the value obtained by multiplying the daily mean of cigarettes smoked by the number of years as a smoker, divided by 20 cigarettes/pack.
- Exclusion criteria:
  1. Any evidence of previous respiratory disease diagnosis (e.g., COPD, chronic bronchitis, asthma, bronchiectasis, etc.).
  2. Pulmonary function examination within previous 12 months,
  3. Any chronic or terminal disorder that, in the researcher’s opinion, could affect the baseline parameters or the tests required for the study,
  4. Impossibility of follow-up participation for any reason, and
  5. Patient refusal to participate or withdrawal from the study.

### Sample calculation

To achieve the study objective, a sample of 1000 participants, 500 in each study group (control and intervention), will be recruited. This will allow the detection of differences in smoking abstinence greater than or equal to 5 %, with 95 % CI and 80 % strength in two-tailed tests, assuming an abstinence rate of 5 % to 6 % in the control group at 12 months and approximately 10 % loss rate during follow-up [24].

### Recruitment, randomization, and data collection (visit 0)

During the selection visit (visit 0), all eligible patients will be advised that smokers are susceptible to various diseases related to tobacco use and smoking cessation will be recommended. They will then be told about the study and offered the possibility of participating. Those who agree will be asked to provide signed informed consent and all the necessary data will be collected using an ad hoc questionnaire that includes:

- Personal and sociodemographic data (age, sex, marital status, and education level and occupation as markers of social class)
- History of diseases, comorbidities, and drug treatments
- History of respiratory symptoms
- Lifestyle (alcohol use and physical activity)
- Smoking habit, current and cumulative (packs/year); nicotine dependence (Fagerström test [29]), motivation to quit smoking (Richmond test [30]), and stage of change (precontemplation, contemplation, preparation, action, maintenance and relapse, according to Prochaska and DiClemente [31])
- Previous quit attempts (number and therapeutic resources used)
- Basic physical examination (weight, height, body mass index, blood pressure)
- eCO levels, measured by MicroCO™ (Micro Medical Ltd, Rochester, UK); this model detects an eCO concentration range of 0-100 parts per million (ppm) with a sensitivity of 1 ppm.

All health professionals working in the 20 primary care centers included in this study will be eligible to participate in the selection process.

#### Randomization

After the selection visit, included participants will be randomized 1:1 to the intervention or control group. The group assignment will be blinded, consecutive, and centralized at the Tarragona Research Support Unit of the IDIAP Jordi Gol, and will follow a simple randomization numeric sequence compiled for this purpose. Of course, due to the nature of the intervention, researchers and participants cannot be blinded to this assignment.

#### Data collection

Individuals assigned to the intervention group will be contacted to return to their primary care center for an evaluation of their pulmonary function using forced spirometry. The test will be carried out by selected nursing personnel with the appropriate technical skills accredited by the Health Studies Institute of the Catalan government, all using the same standard model of spirometer (DATOSPIR-600, SIBEL SA, Barcelona, Spain) with a disposable LILLY-type transducer and following a standard procedure according to ATS/ERS (American Thoracic Society/European Respiratory Society) recommendations [32].

At least three tests will be carried out, from which the best values will be selected for forced vital capacity (FVC), expiratory flow in the first second (FEV1), and ratio of the two measures (FEV1/FVC). Variability between these two tests should be <5 %, expiration time >6 seconds, and the start of the test should show an appropriate and rapid increase. Finally, the reversibility of a possible airway obstruction will be evaluated by a repeat spirometry test 20-30 minutes after the administration of 400 micrograms of salbutamol using a space chamber. All of the tests will be submitted to a single observer, who will centralize quality control and interpret the results using computer software.

### Description of the intervention (visit 1)

All participants will be contacted for an intervention (visit 1) that will include a 5-minute session with their doctor, who will provide health education and advice about quitting smoking, following the usual recommendations for primary care professionals provided by the Tobacco Study Group of the Catalan Society of Family Medicine and the special program, “Atenció Primària Sense Fum” (in Catalan; “Smoke-Free Primary Care”), as shown in Table 2. In addition, they will receive information about the ill effects of tobacco on pulmonary function, illustrated by the Fletcher diagram showing how age-related loss of lung capacity can be accelerated in smokers and how this deterioration slows when one quits smoking, although lost capacity is not recovered [33].

**Table 2:** Summary of the RESET study control and intervention protocols

Control group: brief anti-smoking intervention	Intervention group: spirometry report
In a 5-minute intervention, the health professional will make a clear, firm, personalized proposal recommending smoking cessation, in an empathic and respectful manner. He or she will clearly explain to the smoker that the most important decision the individual can make to achieve better health is to quit smoking, and will provide written informational materials that describe the benefits of giving up smoking.	In a 15-minute intervention, the health professional will carry out an intervention with the same content as the brief anti-smoking intervention and will provide personalized information about the spirometry results, clearing up any patient doubts about spirometry or any other questions that come up during the visit.
The materials are provided by the “Smoke-Free Primary Care” program of the Catalan Society of Family Medicine and the Public Health Agency of Catalonia and are regularly used in primary care offices for brief anti-smoking interventions.	If spirometry values are within normal range, the patient will be informed that his or her pulmonary function has not yet deteriorated and that this would be a good time to quit smoking.
	If spirometry values indicate airway obstruction (FEV1/FVC <70 %), the patient will be informed that he or she could have chronic obstructive pulmonary disease due to smoking, and that the most important treatment measure is to quit smoking.
	If spirometry values show airway restriction, the patient will be informed that his or her pulmonary function could be affected and will be advised to continue with the pulmonary tests normally administered in that primary care center.
	In addition, the patient will be informed of his or her lung age (i.e., the mean age of a nonsmoker with the same FEV1) compared to his or her chronological age, in order to illustrate the possible deterioration of the lungs as a result of smoking.

In addition, participants assigned to the intervention group will receive standardized information about their spirometry results in a personalized visit lasting about 15 minutes, explaining the content of the report in detail. The commentary on each spirometry test will be prepared from a consensus interpretation by the research team, and will focus on a structured description of the results obtained and their interpretation with reference to a theoretical normal value. In addition, the participant will be informed about the “lung age index”, defined as the mean age of a nonsmoker with the same FEV1 value), compared to the chronological age of the participating smoker. This will illustrate the pulmonary deterioration that occurs as a consequence of smoking tobacco [34]. Patients with FEV1 and/or FVC values <80 % of the theoretical reference value and/or FEV1/FVC <0.7 will be informed and advised to undergo the medical testing required to confirm a possible problem with pulmonary function.

Independently of study group assignment, all participants who indicate a desire to quit smoking will be offered the possibility of attending the quit-smoking consultations that are integrated into the everyday activities at each center.

### Follow-up period (visits 2-3)

All participants will be periodically evaluated by their regular primary care team. This will include two follow-up telephone calls, at 3 months (visit 2) and 6 months (visit 3) postintervention, to determine whether they have quit smoking or have made changes in their smoking habits. New data will be collected on current consumption or quit date, if that is the case.

Finally, an in-person visit will be scheduled with each participant (visit 4) after 12 months have passed since study inclusion. At this last study visit, data will again be collected about smoking habits (current consumption, nicotine dependence, motivation and stage of change), attempts to quit, and resources and drug therapy used during the 12-month follow-up. Participants who report that they have quit smoking will be asked to provide their quit date, and their non-smoking status will be confirmed by measuring eCO levels and urine cotinine.

### Definition of variables

#### Outcome measures

The primary outcome variable will be prolonged abstinence at 12 months postintervention. The secondary variable will be point-prevalence abstinence at the end of follow-up. The criterion used to establish the type of abstinence will follow the recommendations of the Society for Research on Nicotine and Tobacco [35]:

- Prolonged abstinence, defined as sustained abstinence from an initial period in which smoking is not counted as a failure (the recommendation is that this period not exceed 2 to 4 weeks) until a follow-up point.
- Point-prevalence abstinence refers to abstinence during a time window immediately before the follow-up point (usually 7 days).

Self-reported smoking abstinence will be validated by biochemical testing at the end of follow-up. Only those participants who report that they quit smoking and have eCO levels less than 10 ppm and urine cotinine values less than 100 ng/mL will be considered nonsmokers [36].

#### Independent measures

The predictive variables to be analyzed include the following:

- Demographic: age, sex, and social class (education level and occupation)
- Anthropometric (weight and height) and main spirometry parameters (FVC, FEV1, and FEV1/FVC%)
- History of comorbidities and risk factors associated with tobacco use (hypertension, diabetes mellitus, ischemic heart disease, cerebrovascular accident, peripheral arterial disease, etc.)
- Lifestyles and habits (physical activity, use of alcohol or other drugs)
- Characteristics of the smoking habit (accumulated and current consumption, stage of change [Prochaska], score on dependency test [Fagerström], and motivation [Richmond]), previous quit attempts and reasons for relapse, and use of drug therapy or other methods to quit smoking during the year of follow-up.

### Data recording and storage

All of the information from the study will be recorded consistently on an ad hoc questionnaire designed for this purpose. Each participant will be assigned a personal identification code upon inclusion, consisting of the initials of their surnames, sex, and birthdate. All information obtained will be stored using an online application accessible only from the Intranet of the Catalan Health Institute in Tarragona. Access to this site is restricted and will be controlled by a personal password for each investigator, who will be responsible for data entry for all of the participants he or she recruits. Weekly back-ups will provide two secure copies of all stored data.

### Statistical analysis

Data for the present study were extracted from a centralized database and grouped so that the person responsible for their analysis was blinded to study group assignments. First, the database was cleansed by detecting and labeling outlier, missing, and inappropriate values. Next we tested the effectiveness of the randomization by evaluating the comparability and homogeneity of the study groups to ensure similar distribution of the variables of interest at baseline. Finally, the proportion of participants lost to follow-up in each group was determined and tested for any association with the study intervention. All analysis was based on intention to treat, understood as having recorded relevant data on the primary variable at the participant’s first visit. Worst-case analysis was applied to assess potential bias due to losses to follow-up [37], which considers that the desired outcome was obtained by all participants lost to follow-up who were in the control group and by none of those lost from the intervention group. Having made this assumption, we repeated the analysis and, to determine the variability associated with the losses to follow-up, compared the “worst-case” results and the results obtained when those lost to follow-up were not taken into account.

Descriptive analysis of quantitative and qualitative variables included frequencies, central tendencies (mean or, in the case of non-normal distribution, median) with standard deviation, and minimum and maximum values. Frequencies were compared using chi-square test or Fisher exact test, as appropriate, and means using Student t test for independent samples.

To evaluate the effectiveness of the smoking cessation intervention, rates of punctual and prolonged abstinence were assessed at 12 months post-intervention in both groups using the chi-square test. The results were presented as relative risk (RR), relative risk reduction (RRR) and absolute risk reduction (RAR) and number needed to treat (NNT) to achieve prolonged smoking abstinence in one smoker, expressed with their 95 % confidence interval (CI). To study the behavior of both groups over time, log-rank tests were used to compare the cumulated abstinence curves over the 12-month follow-up. Finally, multivariate analysis using Cox regression was applied to study the factors associated with smoking cessation success at 12 months. Various models were formulated that included the variable of interest (the described interventions) and as control variables the factors shown to be most significant in the literature [38]: age, sex, social class, employment status, educational level, daily and cumulated tobacco use, degree of nicotine dependence, degree of motivation to quit smoking, and stage of change.

All analysis was done using SPSS version 19.0. Significance was set at *p* < 0.05.

### Limitations of the study

The study has several potential limitations that must be taken into consideration. The first cluster is related to the population. Participants were volunteers recruited when they visited their primary care team for any reason; therefore, they might not be representative of the population of smokers in the primary care system. In addition, those who agreed to participate could be more motivated to quit smoking than the rest of the population of smokers. In any case, the proposed randomization should ensure comparability between study groups. As patients will be recruited by a health care professional with whom they have an existing relationship, participation is expected to be high, especially considering that no drug therapies or invasive or unpleasant tests are involved. Nonetheless, a certain percentage of losses to follow-up is to be expected because this is a long-term (12-month) project. To minimize this contingency, sustained effort by all participating researchers will be essential to motivating and encouraging patients.

With respect to limitations of the study design, the most obvious is that a double-blind study is impossible; both participants and investigators will necessarily be aware of the study group assignments. The proposed intervention may be less intensive than has been described in other studies, but reflects the usual attention received by smokers and therefore can be readily incorporated into the daily clinical practice of the investigators. The content and duration of the intervention have been standardized and participating doctors and nurses will receive specific training to ensure that similar messages, attitudes, and behaviors will be applied in interactions with both groups. In the spirometry intervention group, patient collaboration and technical considerations will be key to achieving quality results. To maximize quality, the testing will be standardized by the use of high-quality pneumotachography and by training of study personnel that results in official accreditation. In addition, to reduce variability in the interpretation of results, data analysis will be centralized.

### Research ethics and confidentiality

The study protocol was approved by the Clinical Research Ethics Committee of IDIAP (Institut Universitari d’Investigació en Atenció Primària) Jordi Gol (registration number 4R11/037) and peer-reviewed by the same experts organization. This trial also has been registered with ClinicalTrial.gov (NCT02153047) on 28/05/2014.

The principal investigator will ensure that the present study is carried out in conformity with all pertinent national and international legislation and with the principles of the Declaration of Helsinki and the Guidelines for Good Clinical Practice published by the Catalan Institute of Health.

All the investigators will use the same standardized protocols, written manuals, specific guidelines, and materials to train health-care personnel to deliver uniform intervention. The protocol will be explained in a training session for all the investigators, and the coordinating center will clarify any questions and problems arising in the course of the study follow-up.

All participants will sign an informed consent prior to participation in the trial, and will receive general information about the study, research objectives and activities included in study participation, number of primary care visits, testing that will be done, information about the results, etc. At all times, data confidentiality will be guaranteed in accordance with Spanish law on personal identity and data, both during the study and in the publication of study results. The instructions provided by the Catalan Institute of Health with respect to access to clinical information for research purposes will also be followed. Documentation will be stored securely and will be available only to study personnel who are authorized to have access.

## Discussion

Data from clinical trials show that increased motivational tension can be a catalyst that leads a smoker to begin or maintain smoking abstinence [39]. However, not all smokers are sufficiently motivated to give up this habit. They may be afraid of the side effects of abstinence (such as weight gain or increased stress), or think they are unable to achieve it (if they have a history of previous relapses), or may simply have no information about the harmful effects of tobacco or the benefits of quitting. In these cases, the patient might be likely to respond to a motivational strategy [5].

Health problems are an important motivational factor to quit smoking and the quit smoking rates seem to be higher in smokers who fear the health complications related to smoking [12]. In this context, the evaluation of individual risk using biomedical tests or assessment of genetic susceptibility to cancer could help to improve abstinence rates [25]. These interventions are based on the idea that informing the smoker about individual risk, based on personal data, could improve understanding of the adverse health effects of smoking and increase motivation to quit smoking [9]. In addition, an individualized focus using risk assessment tools should allow a more effective approach, primarily in smokers with the highest risk and greatest motivation to quit smoking.

This strategy is not new and has been used successfully for years in the prevention of coronary diseases [40]; therefore, in theory it could also apply to a smoking habit. However, the authors of the *Cochrane Review* article that evaluated the effectiveness of informing smokers about biomedical risks as a way of helping them quit smoking concluded that few tests are available and no definitive recommendations can be established [5]. This is a logical conclusion, taking into account the small sample size of most of the 15 studies included in the review article, their methodological heterogeneity, and the lack of benefit detected in each individual study. Only two trials observed significant effects: Parkes et al [23] used reinforcement with spirometry results in terms of “lung age” (RR 2.12; 95 %CI: 1.24-3.62) and Bovet et al [41] showed participants echocardiographically obtained photographs of coronary artery plaque (RR 2.77; 95 %CI: 1.04-7.41). The same review indicates that it is possible to improve the methodological quality of future studies by adjusting the sample size to the research objectives, improving the randomization and the process of making random, blinded assignments, using the consensus definitions of “smoker” and “abstinence”, a biochemical test to confirm smoking abstinence, and an intention-to-treat statistical approach.

The evaluation of pulmonary function in asymptomatic smokers is a controversial topic [20]. Although the use of spirometry has become generalized in our setting, its usefulness in managing COPD in the initial phases of the disease has been questioned because quitting smoking is the most important measure that can be taken to reduce morbidity and mortality; therefore, smoking cessation is an intervention that should always be applied, independently of a COPD diagnosis [42]. Although some observational studies have indicated that early diagnosis of COPD could increase smoking abstinence rates [16, 19], the effectiveness of spirometry as a motivational tool has not yet been demonstrated convincingly in a clinical trial [43]. To the contrary, some doubts have been raised and there have been suggestions that providing information about a normal result could have a “tranquilizing effect” that strengthens the bias of optimism that some smokers have about their own smoking habit [44]. In any case, and in light of the available data, it is evident that more research is needed to determine whether feedback on spirometry data has a differential impact on smokers depending on the evidence of deteriorated pulmonary function.

In this context, the RESET is proposed as a randomized, controlled, parallel, multicenter clinical trial that will include smokers with no known history of respiratory disease, with the primary objective of evaluating whether an intervention based on proving information about spirometry as a motivational element will improve the success rates of smoking cessation interventions in primary care settings. Perhaps the results of the proposed study will contribute to clarifying some of the uncertainties that currently exist.

## Abbreviations

ARR: absolute risk reduction
ATS/ERS: American Thoracic Society, European Respiratory Society
CI: confidence interval
CO: carbon monoxide
COPD: chronic obstructive pulmonary disease
eCO: exhaled carbon monoxide
FEV1: forced expiratory volume in the first second
FVC: forced vital capacity
ICH: International Conference on Harmonisation
IDIAP: Primary Care Research Institute
NNT: number needed to treat
OR: odds ratio
ppm: parts per million
RR: relative risk
RRR: relative risk reduction
